# Typhoid outbreak investigation in Dzivaresekwa, suburb of Harare City, Zimbabwe, 2011

**DOI:** 10.11604/pamj.2014.18.309.4288

**Published:** 2014-08-18

**Authors:** Monica Muti, Notion Gombe, Mufuta Tshimanga, Lucia Takundwa, Donewell Bangure, Stanley Mungofa, Prosper Chonzi

**Affiliations:** 1 Department of Community Medicine, University of Zimbabwe, Harare, Zimbabwe; 2 Harare City Health Department, Harare, Zimbabwe

**Keywords:** Typhoid, outbreak investigation, contaminated water, Zimbabwe

## Abstract

**Introduction:**

Typhoid fever is a systemic infection caused by a Gram negative bacterium, *Salmonella typhi*. Harare City reported 1078 cases of suspected typhoid fever cases from October 2011 to January 2012. We initiated an investigation to identify possible source of transmission so as to institute control measures.

**Methods:**

An unmatched 1:1 case-control study was conducted. A questionnaire was administered to study participants to identify risk factors for contracting typhoid. A case was a resident of Dzivaresekwa who presented with signs and symptoms of typhoid between October and December 2011. Water samples were collected for microbiological analysis.

**Results:**

115 cases and 115 controls were enrolled. Drinking water from a well (OR= 6.2 95% CI (2.01-18.7)), attending a gathering (OR= 11.3 95% CI (4.3-29.95)), boiling drinking water (OR= 0.21 95% CI (0.06-0.76)) and burst sewer pipe at home (OR= 1.19 95% CI (0.67-2.14)) were factors associated with contracting typhoid. Independent risk factors for contracting typhoid were drinking water from a well (AOR = 5.8; 95% CI (1.90-17.78)), and burst sewer pipe at home (AOR = 1.20; 95% CI (1.10-2.19)). Faecal coli forms and E. coli were isolated from 8/8 well water samples. Stool, urine and blood specimens were cultured and serotyped for Salmonella typhi and 24 cases were confirmed positive. *Shigella, Giardia and E coli* were also isolated. Ciprofloxacin, X-pen and Rocephin were used for case management. No complications were reported.

**Conclusion:**

Contaminated water from unprotected water sources was the probable source of the outbreak. Harare City Engineer must invest in repairing water and sewage reticulation systems in the city.

## Introduction

Typhoid fever is a life-threatening epidemic prone disease caused by a gram negative bacterium, *Salmonella* Typhi (*S*. Typhi). Humans are the only natural host and reservoir of the bacteria. Persons with typhoid fever carry the bacteria in their bloodstream and intestinal tract [1, 2]. Typhoid fever is spread from person to person through the fecal-oral route, by ingestion of food or water contaminated with faecal matter. *S*. Typhi may also be found in vomitus and urine. Polluted water is the most common source of transmission. The incubation period is usually 8 - 14days but can range from 3 days to 2 months. The attack rate and incubation period are greatly influenced by the size of the innoculum and type of vehicle in which the food is ingested [1–4].

In January 2010 Zimbabwe witnessed a resurgence of typhoid fever in Mabvuku and Tafara suburbs of the City of Harare. This was against a backdrop of challenges in sewage reticulation and access to safe water. Harare City experienced another typhoid outbreak from October 2011to June 2012. The first positive case was confirmed by laboratory diagnosis, on the 31^st^ of October confirming a typhoid outbreak. The 2^nd^ case was confirmed on the 1^st^ of November. This study was conducted to investigate the factors associated with contracting typhoid in Dzivaresekwa which was the epicentre of the outbreak. Specifically we intended to describe the outbreak by person, place and time, identify risk factors associated with contracting typhoid, assess knowledge of typhoid in the affected community, assess case management and assess the outbreak preparedness and response by the city health department.

## Methods

An unmatched 1:1 Case Control study was conducted in Dzivaresekwa suburb of Harare City. A case was defined as any person from Dzivaresekwa presenting with any one or more of the following symptoms: fever for more than 3 days, associated with malaise, headache, vomiting, diarrhea or constipation and cough from the 9^th^ of October 2011 to the 12^th^ of December 2011. A control was a neighbour of a case who did not suffer from typhoid symptoms during the period under study. Cases were selected from the line list using random a numbers. An interviewer administered questionnaire was used to collect data on the factors associated with contracting typhoid as well as the knowledge, and practices regarding typhoid. A check list was used for the environmental and home assessment of the respondents and to assess the outbreak preparedness and response of the Harare City Health Department. Records of patient notes were also reviewed to obtain information on case management. Stool specimens were collected at end of treatment to establish the proportion of patients who remained carriers at end of treatment. Knowledge was assessed using multiple choice questions with each correct response being worth one point. A participant's knowledge score was the total number of points earned out of 15 points. Total knowledge scores were classified as “good” (>80%), “fair” (70-79%), or “poor” (<69%) based on cut off points from an evaluation of knowledge, attitudes, and practices of health care providers toward HIV-positive patients in Tanzania.

## Results

A total of 115 cases and 115 controls were interviewed. The median age of the cases was 18 years (Q_1_ =7, Q_3_ =29) and the median age for controls was 30 (Q_1_ = 25, Q_3_ =39). More female controls were unemployed (56.5%) compared to cases (27.8%). No minors were interviewed among the controls but for those cases who were minors, their parents or guardians were interviewed. The majority of the cases (30%) were students while the majority of controls (56.5%) were unemployed. For the case, 52.2% had attained a secondary education while 71.3% of the controls had attained a secondary level education.Table 1 shows the demographic characteristics of the cases and the controls.

**Table 1:** Demographic characteristics of the cases and controls, Dzivaresekwa Suburb October 2011 - December 2011

Attribute	Cases n (%)	Controls n (%)	p-value
**Sex**			0.000
Male	59(51.3)	33(28.7)	
Female	56(48.7)	82(71.3)	
**Occupation**			0.001
Formally Employed	10(8.7)	9(7.8)	
Minor	23(20.0)	0(0.0)	
Self Employed	15(13.0)	37(32.2)	
Student	35(30.4)	4(3.5)	
Unemployed	32(27.8)	65(56.5)	
**Education**			0.000
Never been to school	24(20.9)	3(2.6)	
Primary School	29(25.2)	21(18.3)	
Secondary	60(52.2)	82(71.3)	
Special School	1(0.90)	0(0.0)	
Tertiary	1(0.9)	9(7.8)	

**Figure 1:**
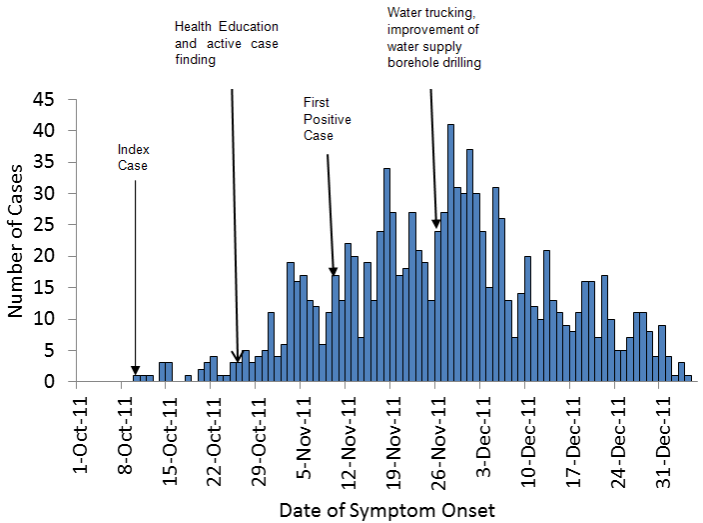
Harare City Health Typhoid Outbreak Epicurve: October 2011 to January 2012

Twenty four cases were found to be positive for *Salmonella typhi* in stool, urine or blood. Only one death was reported. For the under fives, males were more affected (30%) than females (22%) while for the rest of the age groups females were more affected. Of all the age groups, the 25 to 44 age group was most affected with 29% male and 30%female cases. Figure 1 shows the epidemic curve which indicates a propagated outbreak. Approximately two weeks after the improvement in municipal water supply and drilling of boreholes, the number of cases showed a decline.

Drinking water from a well (OR= 6.2, 95% CI (2.01-18.7)), attending a gathering (OR= 11.3 95% CI (4.3-29.95)), boiling drinking water (OR= 0.21 95% CI (0.06-0.76)) and burst sewer pipe at home (OR= 1.19 95% CI (0.67-2.14)) were factors associated with contracting typhoid. The relationship between having a typhoid contact at home and contracting typhoid was modified by gender. Females were 19.5 times more likely to contract typhoid if there was a typhoid contact at home while males had a lower risk.

Independent risk factors for contracting typhoid were storing water in wide mouthed containers with lid (AOR 3.68 (1.62-8.35)), having a typhoid contact at home (AOR= 8.34, 95%CI (3.82 - 18.18)), drinking water from a well (AOR = 5.8; 95% CI (1.90-17.78)) and burst sewer pipe within 500m of household (AOR = 1.20; 95% CI (1.10-2.19)). Storing water in narrow mouthed containers with lid (AOR = 0.43, 95%CI (0.22-0.80)), availability of a rack to dry plates. (AOR 0.3, 95% CI (0.1-0.5)) and boiling drinking water (AOR = 0.24, 95%CI (0.07-0.90)) were protective. Table 2 shows the factors associated with contracting typhoid.

**Table 2:** Independent factors associated with contracting typhoid in Dzivaresekwa, Harare, 2011

Factor	Cases n (%)	Controls n (%)	AOR (95%CI)
Water from well	21(18.3)	4 (3.5)	5.8 (1.90-17.78)
Burst sewer pipe within 500m of home	33 (28.7)	29(25.2)	1.2 (1.10-2.19)
Typhoid contact at home	48(41.7)	10(10.5)	8.34 (3.82 – 18.18)
Store water in wide mouthed container with lid	102(89.6)	90(73.9)	3.68 (1.62-8.35)
Boil drinking water	3 (2.6)	13(11.3)	0.24 (0.07-0.90)
Storage of water in a narrow mouthed container with lid	19(16.5)	40(34.8)	0.43 (0.22-0.80)

**Knowledge on typhoid:** Knowledge on typhoid was poor among both cases and controls. None of the controls had a good knowledge of typhoid while only 3(2.6%) cases had a good knowledge. Knowledge of the cause of typhoid was especially poor with only 5.2% cases and 7.8% controls mentioning that it is caused by germs. The majority of both cases and controls (68.7% and 67.8% respectively) thought typhoid is caused by drinking unsafe water. Cases had more knowledge of the symptoms of typhoid compared to controls. More cases (56.5%) than controls (36.5%) knew that fever is a symptom of typhoid (p = 0.001). There was no significant difference however in knowledge of the other symptoms between cases and controls. There was a significant difference between cases and controls in knowledge that eating contaminated food helps in the spread of typhoid (p = 0.016).

**Environmental assessment:** There was a clean toilet in homes of 83.5% of cases and 88.7% of controls. Eighty five percent of the cases had soap available compared to 90.4% of the controls while 25.2% of cases versus 38.3% of controls had a functional flush toilet.

**Water testing:** Samples of water were collected from shallow wells, boreholes and municipal taps for microbiological analysis. On 3 consecutive occasions, two of the 6 boreholes sampled were positive for *E. coli* and faecal coliforms. All shallow wells were positive for *E. coli* and faecal coliforms. All municipal tap water samples (10/10) were negative for faecal coliforms and *E. coli*. All the well water samples (8/8) were positive for *E. coli* and fecal coliforms. A sample from a spring yielded the same microorganisms. Treated shallow well water had residual chlorine of 0.1 mg/litre while 14 samples from municipal tap water had residual chlorine ranging from 0.1 - 0.5mg/litre.

**Case Management:** The most common symptoms suffered by the interviewed cases were fever (76.5%), headache (68.7%), diarrhea (67.8) and abdominal cramps (61.7%). Ciprofloxacin, X-pen and rocephin were used for case management based on drug sensitivity results. Nalidixic acid syrup was used for management of paediatrics about 2weeks into the outbreak. It was however discontinued after concerns of inability to achieve optimum blood concentrations as well as the probability of promoting decreased susceptibility or full blown resistance to ciprofloxacin. Intravenous fluids and oral rehydration solution were used as needed. No complications were reported. Patients were discharged from hospital on average after 3 days. Stool specimens were collected from those who tested positive for *S. typhi* to establish whether or not they continued to shed the salmonella typhi.

**Outbreak response:** The district response was good in terms of notification, concrete response and, completing of line lists. However there was delay in reporting of the index case to the health facility and there was need to improve on the timeliness of updating line lists. The health team was alerted on the possibility of an outbreak on the 25^th^ of October 2011. Active case finding thus began together with contact tracing. Door to door health education on typhoid prevention and control was started in Dzivaresekwa, initially by Health Promoters (HPs) from the city health department and was later complemented by partners. Water samples were taken from shallow wells, boreholes and municipal taps. Sewer bursts were also found to be a constant phenomenon. Water storage containers and soap were distributed to 3000 households. A three month supply of water treatment chemicals for point of use was distributed in all affected areas. Water trucking was conducted and 6 boreholes were drilled. Burst sewer pipes were repaired or replaced. Municipal water supply was improved almost a month after the outbreak had been declared. Water trucking and drilling of boreholes were put in place about three weeks into the outbreak.

## Discussion

In this study, poor sanitation and water supply were the major factors associated with contracting the disease. This is consistent with a study by Ram et al which showed that sanitation and water play a central role in the transmission of typhoid [5]. The use of wide mouthed containers for storing water was a risk factor to contracting typhoid and this is biologically plausible given the likelihood of contaminating water when getting it out of the container using another container which may not be clean. Having a typhoid contact was also a risk factor and this is also supported by the epicurve which illustrates a propagated outbreak, an indication of person to person spread. Recent typhoid contacts were also found to be significantly associated with typhoid fever in a study in Indonesia. In this Indonesian study, crowding (defined as >6 household members) was also associated with contracting typhoid fever [6]. Though in our study we did not compare household sizes between cases and controls, it was noted during household visits and environmental assessments that at the epicenter of the outbreak there was crowding. More than two households were noted to be residing in one housing unit. This, coupled with poor water supply and poor sanitation, could have fueled the outbreak.

The relationship between having a typhoid contact at home and contracting typhoid was modified by gender. These results are consistent with results from a study by Madembo et. al. on factors associated with contracting typhoid in Mabvuku suburb of Harare. Females tend to be caregivers of those who are ill at home so if someone fell ill with typhoid, they were more likely to have more contact with females in the household as they were cared for during the illness. Thus there was higher likelihood of spreading the disease if appropriate hygiene practices were poorly observed.

In our study, all shallow wells were positive for *E. coli* and faecal coliforms but this was not surprising as the shallow wells are downstream from sewer flows when there are sewer blockages. At the epicenter of the outbreak, in Dzivaresekwa 3, there was very erratic water supply. In some households there was no water supply at all either because of water cuts due to non-payment or clogged pipes due to ageing. As a result residents did not have potable water for household use and resorted to digging shallow wells in the vicinity of their households. However these wells were prone to contamination with flowing sewage, thus the poor water quality. Another study by Gasem et al. showed that low socioeconomic status, poor housing with inadequate water supply and open sewers and inappropriate personal hygiene were associated with increased risk of contracting typhoid [5].

Washing hands was found to be a protective though not significant factor against contracting typhoid in the bivariate analysis. This was probably because both cases and controls had received door to door education on hygiene and sanitation. Therefore they had probably improved their hygiene practices at the time of the study, thus there was no significant difference between the cases and controls. Washing hands after using the toilet and before eating remained protective after multivariate analysis though not significant.

Only 78% of the cases interviewed reported or had a record of fever as one of the symptoms they were suffering. This is contrary to the fact that fever was one of the symptoms that was used for screening patients at Rujeko Clinic. Thus there was poor recording of the symptoms. Records review also showed discrepancies in symptoms recorded on the line list and those recorded on the case notes kept at the referral hospital. More female controls were interviewed compared to males. This is because females were more likely to be at home when interviewer visited the household. More cases than controls knew the symptoms of typhoid probably because they had suffered one or more of the symptoms. The lack of significant differences in knowledge of most of the variables could be due to the door to door education conducted during the outbreak.

For the diagnosis of typhoid, blood culture is the standard diagnostic test provided a large amount of blood is collected (15ml in adults). Blood culture is positive in 60-80% of typhoid patients and its sensitivity is higher in the first week of infection. Stool culture sensitivity depends on amount of stool cultured and positivity increases with duration of illness [7]. At the health centre level, Rujeko clinic, stool specimens were being collected from both cases and contacts. A transport system was put in place where specimens were collected 3times a day from the clinic to the laboratory. There was however concern on the possibility of delay in transporting specimens to the laboratory resulting in low isolation rate (24 *S typhi* positives out of 1078 suspected cases). There was a possibility that we could be missing some positives. Training of for nurses, doctors and environmental health officers in specimen handling would help to enable proper collection and handling of specimens.

Strong evidence exists that fluoroquinolones are the most effective drugs for treatment of typhoid fever, with cure rates exceeding 96%. The review on typhoid by Parry et. al also showed that third-generation cephalosporins (ceftriaxone, cefixime, cefotaxime, and cefoperazone) and azithromycin are also effective drugs for typhoid^7^ In our study, cases were being managed with ciprofloxacin and ceftriaxone as the antibiotics of choice based on drug sensitivity results. Benzyl Penicillin and paracetamol were administered based on need. Other drugs used include erythromycin and cotrimoxazole. The use of these other antibiotics was for patients suspected of other infection like TB where it would not be appropriate to use ciprofloxacin. Concerns were also raised on the poor link with private practitioners where chloramphenicol was used in management of one patient by a private practitioner. Drug sensitivity results had indicated resistance to chloramphenicol.

While response by the health team in attending to the outbreak was swift, there was a delay in response regarding water supply during the outbreak. Boreholes were drilled approximately 3 weeks into the outbreak. Even then municipal water supply had not improved. It was however noted that this was probably due to poor coordination with other relevant stakeholders thus they did not have an appreciation of the role potable water supply could have in this outbreak. The availability of an Epidemic preparedness and Response team which includes all relevant stakeholders would have helped to speed up response. This team should have regular updates during or outside outbreak situations so that they are prepared for outbreaks and can react swiftly.

The other major challenge was also the fact that the city cannot meet the demand for water thus the erratic supply of potable water in parts of the city. The engagement of relevant stakeholders by the Town Engineer for a long term solution in water supply to meet the growing demands of the capital city is recommended.

It took 11 days between the onset of symptoms of the index case to the time the first outbreak case came to the health facility. The index case did not get to the health facility until after she was found to be positive for S typhi during contact screening. Even then, the first few cases did not seek health care until they were visited by environmental health officers. This depicts poor health seeking behaviour on the community in question. One of the probable reasons could be poverty which makes them unable to pay for health services at the local clinic.

## Conclusion

In conclusion, poor water and sanitation was associated with the typhoid outbreak. Boiling water and the use of narrow mouthed water containers for water storage are recommended. The City Engineer must replace old sewer and water reticulation pipes.
